# Increased Plasma Heme Oxygenase-1 Levels in Patients With Early-Stage Parkinson’s Disease

**DOI:** 10.3389/fnagi.2021.621508

**Published:** 2021-02-12

**Authors:** Wenhua Sun, Jinhua Zheng, Jianjun Ma, Zhidong Wang, Xiaoxue Shi, Mingjian Li, Shen Huang, Shiyu Hu, Zhenxiang Zhao, Dongsheng Li

**Affiliations:** ^1^Department of Neurology, People’s Hospital of Zhengzhou University, Zhengzhou, China; ^2^Department of Neurology, Henan Provincial People’s Hospital, Zhengzhou, China; ^3^Department of Neurology, People’s Hospital of Henan University, Zhengzhou, China

**Keywords:** biomarker, heme oxygenase-1, Parkinson’s disease, plasma, voxel-based morphometry

## Abstract

**Introduction**: Heme oxygenase-1 (HO-1) is a 32 kDa stress-response protein implicated in the pathogenesis of Parkinson’s disease (PD). Biliverdin is derived from heme through a reaction mediated by HO-1 and protects cells from oxidative stress. However, iron and carbon monoxide produced by the catabolism of HO-1 exert detrimental effects on patients with PD. The purpose of this study was to determine whether plasma HO-1 levels represent a biomarker of PD and to further explore the underlying mechanism of increased HO-1 levels by applying voxel-based morphometry (VBM).**Methods**: We measured plasma HO-1 levels using an enzyme-linked immunosorbent assay (ELISA) in 156 subjects, including 81 patients with early- and advanced-stage PD and 75 subjects without PD. The analyses were adjusted to control for confounders such as age, sex, and medication. We analyzed T1-weighted magnetic resonance imaging (MRI) data from 74 patients with PD using VBM to elucidate the association between altered brain volumes and HO-1 levels. Then, we compared performance on MMSE sub-items between PD patients with low and high levels of HO-1 using Mann-Whitney *U* tests.**Results**: Plasma HO-1 levels were significantly elevated in PD patients, predominantly those with early-stage PD, compared with controls (*p* < 0.05). The optimal cutoff value for patients with early PD was 2.245 ng/ml HO-1 [area under the curve (AUC) = 0.654]. Plasma HO-1 levels were unaffected by sex, age, and medications (*p* > 0.05). The right hippocampal volume was decreased in the subset of PD patients with high HO-1 levels (*p* < 0.05). A weak correlation was observed between right hippocampal volume and plasma HO-1 levels (*r* = −0.273, *p* = 0.018). There was no difference in total MMSE scores between the low- and high-HO-1 groups (*p* > 0.05), but the high-HO-1 group had higher language scores than the low-HO-1 group (*p* < 0.05).**Conclusions**: Plasma HO-1 levels may be a promising biomarker of early PD. Moreover, a high plasma concentration of the HO-1 protein is associated with a reduction in right hippocampal volume.

## Introduction

Parkinson’s disease (PD) is a movement disorder characterized by the loss of dopaminergic neurons in the substantia nigra and the formation of Lewy bodies induced by the aggregation of α-synuclein. Although pharmacological dopamine substitution (L-DOPA treatment) or deep brain stimulation (DBS) have been used to control the symptoms of PD and improve the quality of life up to decades after the onset of disease, these treatments are not very effective at preventing PD progression. Therefore, PD is a neurodegenerative disease that may ultimately lead to severe disability as it progresses (Poewe et al., 2017).

To date, the diagnosis of PD has been based on clinical criteria (Hughes et al., 1992). However, more than half of dopaminergic neurons are typically lost before a patient is diagnosed with PD. The long preclinical phase of PD offers the potential to administer disease-modifying treatments; therefore, an easily accessible biomarker that reflects the process of neurodegeneration is needed to diagnose the disease and predict disease progression in individual patients. Thus, a reliable biomarker would have great potential to help distinguish patients with PD from healthy persons, particularly in the early stages of the disease.

Heme oxygenase-1 (HO-1) is a 32 kDa cellular stress protein of the superfamily that metabolizes heme into biliverdin, iron, and carbon monoxide in cooperation with NADPH cytochrome P450 reductase in the brain and other tissues exposed to oxidative stress. This enzyme is primarily located and functions in the endoplasmic reticulum. Biliverdin is then further oxidized to bilirubin by biliverdin reductase, which helps restore a more favorable tissue redox microenvironment (Maines, 1988; Keyse and Tyrrell, 1989). The neuroprotective effect of HO-1 has been well studied in PD. For example, failure of proteasome degradation results in the formation of misfolded proteins, which is an important mechanism of α-synuclein accumulation in PD. Meanwhile, overexpression of HO-1 has been shown to facilitate α-synuclein proteasomal degradation in M17 neuroma cells (Song et al., 2009). Heme-derived CO in glial cells plays an important role in the dopaminergic neuroprotective effect of 2’, 3’-dihydroxy-4’,6’-dimethoxychalcone (DDC) through cell-to-cell communication (Masaki et al., 2017). Simvastatin prevents oxidative stress by enhancing the expression of the antioxidant protein HO-1 in a 6-OHDA-induced PD model (Tong et al., 2018). On the other hand, the following results indicate that overactivation of HO-1 in neurons may be deleterious in PD. Overexpression of HO-1 has been demonstrated in astrocytes in postmortem PD samples (Schipper, 2004). Moreover, excessive upregulation of HO-1 promotes the accumulation of non-transferrin-bound iron deposits, mitochondrial dysfunction, macrophages, and oxidative stress (Schipper, 1999; Song et al., 2006; Zukor et al., 2009). Previous studies have reported that selective overexpression of HO-1 in astrocytes in GFAP.HMOX1 transgenic mice from 8.5 to 19 months of age lead to the behavioral, neuropathologic, and molecular biological characteristics of parkinsonism (Song et al., 2017). Based on these findings, HO-1 may be a double-edged sword, and some clinical studies have investigated this hypothesis. Significantly higher serum (Mateo et al., 2010) and salivary HO-1 levels (Song et al., 2018) have been reported in patients with PD than in normal controls, prompting the hypothesis that HO-1 levels in biological fluids represent a promising marker of PD and may be used to assess the extent of neurodegeneration.

Voxel-based morphometry (VBM) is a widely used automated neuroanatomical image analysis technique that compares the local concentrations or volumes of gray and white matter in voxels (Yousaf et al., 2018). In the past decade, VBM has been widely used in neurological research, including studies of PD (van Mierlo et al., 2015; Goto et al., 2018; Xuan et al., 2019; Wolters et al., 2020). Here, we chose to investigate potential differences in the voxels of brain regions between patients with high and low plasma HO-1 levels (using the mean value as the dividing point) by applying this structural imaging technique at an anatomical level to explore the mechanism of the differences in HO-1 levels in PD patients.

In the present study, we sought to determine by ELISA whether plasma-derived HO-1 protein concentrations are elevated in patients with idiopathic PD and to explore structural neuroimaging to elucidate the mechanism underlying the variability of HO-1 levels in the blood of these patients.

## Materials and Methods

### Participants

All participants were recruited from the Henan Provincial People’s Hospital. This study included 156 participants: 81 patients with PD and 75 healthy controls. After initial inclusion, seven subjects with PD and incomplete relevant imaging data were excluded. The final sample for structural MRI analyses consisted of 74 patients with PD. The inclusion criteria were as follows: (1) a diagnosis of PD was made by an experienced neurologist; and (2) PD was diagnosed according to the United Kingdom Brain Bank Clinical Diagnostic Criteria (Hughes et al., 1992). Patients with secondary parkinsonism or atypical parkinsonian syndromes (including multiple system atrophy, progressive supranuclear palsy, dementia with Lewy bodies, and cortical–basal ganglionic degeneration) and individuals with the systemic inflammatory disease were excluded from the study.

The patients with idiopathic PD were in the “on” period when they were evaluated using different scales. The severity of motor symptoms was evaluated using the Hoehn & Yahr stages (H & Y stages) and United Parkinson’s disease (PD) Rating Scale-III (UPDRSIII; Goetz et al., 2008). Cognition was assessed with the Mini-Mental State Examination (MMSE). The MMSE was divided into eight subitems: (1) orientation to time and place (10 points); (2) immediate registration (three points); (3) divided attention (five points); (4) delayed recall (three points); (5) language (four points); (6) following a three-step command (three points); (7) writing a sentence (one point); and (8) copying a figure (one point; Ringman et al., 2007). The patients were on PD medication, including carbidopa/levodopa and dopamine agonists, to relieve their symptoms; however, exposure to these medications can influence plasma HO-1 concentrations and thereby act as a confounding factor. Also, there are more than a dozen anti-PD medications, and patients with PD usually take two or more medications. We consulted the drug treatment history of the PD patients and used a previously reported formula to calculate the equivalent daily doses of L-DOPA (LEDD; Tomlinson et al., 2010).

Meanwhile, 75 sex-matched subjects without PD were recruited from the Outpatient Physical Examination Department of Henan Provincial People’s Hospital. The inclusion criteria for control subjects were as follows: (1) no history of neurological disease or psychiatric disorders; (2) no brain injury; and (3) no diabetes mellitus, hypertension, cardiovascular disease, or systemic diseases (inflammation, infection, cancer, et cetera). The inclusion and exclusion criteria were strictly enforced to minimize the potential for confounders.

We divided patients with PD into those in the early stages (H&Y stage I and H&Y stage II) and those in the advanced stages (H&Y stage III and H&Y stage IV) to investigate the relationship between plasma HO-1 levels and disease progression. This study was approved by the ethics committee of Henan Provincial People’s Hospital. All study participants and their guardians provided written informed consent. The methodology used in this study was implemented according to recognized guidelines.

### Blood Sampling and Analysis of Plasma HO-1 Levels

Early in the morning (7:00 am) after an overnight fast, a 5-ml blood sample was collected from each participant into a tube containing the anticoagulant EDTA. The blood samples were centrifuged (2,500 *g* for 15 min) at 25°C within 1 h of collection. The supernatant was collected, divided into 1.5 ml centrifuge tubes, and stored at −80°C until analysis. Plasma HO-1 levels in subjects with and without PD were measured using a human HO-1 sandwich ELISA kit (Catalog Number: ADI-EKS-800; Enzo Life Sciences Inc., Farmingdale, NY, USA) according to the manufacturer’s protocol. A standard curve generated with purified HO-1 was used to calculate the HO-1 concentration of each sample from the absorbance at λ = 450 nm (Li Volti et al., 2010). The sensitivity of the HO-1 (human) ELISA kit was determined to be 0.78 ng/ml. The standard curve had a range of 0.78–25 ng/ml. The intra-assay coefficient of variation of the HO-1 (human) ELISA kit was determined to be <10%. All samples were analyzed in duplicate and processed in the first freeze-thaw cycle.

### Neuroimaging Data Acquisition and Preprocessing

All MRI scans were acquired using a Siemens MAGNETOM Prisma 3 T MRI scanner with a 64-channel head coil. Structural images were obtained using a 3D MRI magnetization-prepared rapid gradient-echo (MPRAGE) T1-weighted sequence [matrix = 256 × 256; flip angle = 4°; field of view (FOV) = 256 × 256 mm; slice thickness = 1 mm; TR = 5,000 ms; echo time (TE) = 3.43 ms; voxel size = 1 × 1 × 1 mm].

The quality of the acquired MRI scans was visually assessed to exclude scans with severe vascular lesions, space-occupying lesions, or motion artifacts. VBM was performed with SPM8^1^ running on MATLAB 2013b. T1 image preprocessing was implemented using VBM8^2^ as follows (Chard et al., 2002): (1) format conversion: the DICOM images were converted to a parsable NIFTI format; (2) normalization: after image registration, all anatomical images were registered to the Montreal Neurological Institute (MNI) coordinate space with a voxel size of 1 × 1 × 1 mm^3^; (3) segmentation: the brain tissue was segmented into gray matter (GM), white matter, and cerebrospinal fluid; (4) smoothing: the normalized modulated images were smoothed with an 8-mm full-width at half-maximum (FWHM) Gaussian kernel.

### Whole-Brain and Region-of-Interest (ROI) Analyses

The modulated and smoothed GM data were subjected to whole-brain analysis using a two-sample *t*-test. Two sets of between-group analyses were performed on the differences in GM volume using a two-sample *t*-test in SPM8. Age and sex were included as covariates. First, the whole-brain significance threshold was set to *p* < 0.05 for multiple comparisons using familywise error correction at the voxel level. Since *in vivo* evidence of a correlation between GM and HO-1 levels in patients with PD has never been reported, the data were also presented using a less stringent, uncorrected threshold (*p* < 0.001, cluster threshold = 10 voxels) to detect subtle morphological changes. Based on the results of an exploratory whole-brain analysis, we also performed ROI analyses focusing on the right hippocampus. The brain areas with identified intergroup differences served as ROIs. The XJVIEWER^3^ module was applied to save the ROIs as a mask, and the GM volume of the ROI was extracted using REST1.2^4^. Finally, the results of the SPM8 analysis were visualized using MRICRON^5^ and Adobe Photoshop 2019 (Adobe Systems Incorporated, San Jose, CA, USA).

### Statistics

The normality of distributions was assessed by the Shapiro–Wilk (SW) normality test (Henderson, 2006). Normally distributed continuous data are expressed as the mean ± standard deviation (SD). Data that did not conform to the normal distribution are presented as the median (first quartile, third quartile). Pearson’s chi-square test was used to compare the distributions of categorical variables between groups. Two-group comparisons of normally distributed variables were performed using *t*-tests. One-way ANOVA was performed to compare variables among three or more groups. Variables that did not fit the normal distribution were assessed using the Mann-Whitney *U* test (two groups) or the Kruskal–Wallis (KW) rank-sum test (three or more groups).

Spearman’s rank correlation coefficient was calculated to assess whether HO-1 levels were related to medications, and the correlation between plasma HO-1 levels and ROI volumes was analyzed by calculating the Pearson correlation coefficient.

The accuracy of plasma HO-1 levels in diagnosing patients with PD was assessed by constructing a receiver operating characteristic curve. The point with the maximum Youden index on the ROC curve was considered the optimum HO-1 cutoff point to diagnose the disease.

Statistical analyses were performed using SPSS software version 25 (IBM, USA). Graphs were generated with GraphPad Prism version 8.0 (GraphPad Prism Software, Inc., San Diego, CA, USA). A value of *p* < 0.05 was considered to indicate a statistically significant difference.

## Results

Of the 156 study subjects, 81 (51.9%) were diagnosed with idiopathic PD, and 75 (48.1%) were non-PD controls. The clinical characteristics of patients with PD are summarized in Table 1. No intergroup differences in sex were observed (*p* = 0.985), but patients with PD were older than controls (*p* < 0.001).

**Table 1:** Clinical characteristics between PD group and controls.

	PD (*n* = 81)	Control (*n* = 75)	*p* value
Gender (male/female)	42/39	39/36	0.985
Age (years)	64.00 (57.50, 67.00)	51 (47, 57)	<0.001*
Age of onset (years)	58.00 (50.00, 61.50)	NA	
Disease duration (years)	5.00 (4.00, 9.00)	NA	
Hoehn and Yahr scale	2.00 (2.00, 3.00)	NA	
UPDRS III	38.99 ± 16.37	NA	
LEDD (mg)	400.00 (212.50, 600.00)	NA	
MMSE	26.00 (24.00, 28.00)	NA	

Mean ± standard deviation or median (interquartile range). UPDRS, United Parkinson’s Disease Rating Scale; MMSE, Mini-Mental State Examination; LEDD, The equivalent daily dose of L-DOPA; HO-1, Heme Oxygenase-1; PD, Parkinson’s disease; NA, not available. *p < 0.05.

There were no statistically significant differences in HO-1 levels among the three groups (early PD patients, advanced PD patients and healthy controls) in either men or women [male: 2.39 (2.11, 2.97) vs. 2.38 (2.02, 2.61) vs. 2.18 (1.70, 2.61) ng/ml, *p* = 0.176; female: 2.34 ± 0.60 vs. 2.10 ± 0.53 vs. 2.01 ± 0.41 ng/ml, *p* = 0.053; Supplementary Figure 1]. Furthermore, comparison of HO-1 levels between men and women in the early PD, the advanced PD, and control groups also showed no significant differences [early PD: 2.39 ± 0.75 vs. 2.34 ± 0.60 ng/ml, *p* = 0.81; advanced PD: 2.33 ± 0.58 vs. 2.10 ± 0.53 ng/ml, *p* = 0.22; normal controls: 2.18 (1.70, 2.61) vs. 2.07 (1.74, 2.28) ng/ml, *p* = 0.25; Supplementary Figures 1B–D]. We also performed the same stratified analysis with age as a confounding factor. Subjects aged ≥60 years and < 60 years were defined as elderly and young, respectively. When the levels of HO-1 were compared among the early PD, advanced PD and control groups in young and elderly subjects, the results showed no statistically significant differences [young subjects: 2.43 (1.99, 2.97) vs. 2.19 (1.62, 2.61) vs. 2.16 (1.73, 2.39) ng/ml, *p* = 0.15; elder subjects: 2.34 ± 0.70 vs. 2.23 ± 0.56 vs. 2.02 ± 0.49 ng/ml, *p* = 0.22; Supplementary Figure 1E]. Comparison of HO-1 levels between young and elderly individuals in the early PD, advanced PD, and normal control groups also showed no significant differences [early PD: 2.42 ± 0.62 vs. 2.34 ± 0.70 ng/ml, *p* = 0.75; advanced PD: 2.19 ± 0.60 vs. 2.23 ± 0.56 ng/ml, *p* = 0.84; normal controls: 2.16 (1.73, 2.39) vs. 2.09 (1.68, 2.33) ng/ml, *p* = 0.69; Supplementary Figures 1F–H].

**Figure 1:**
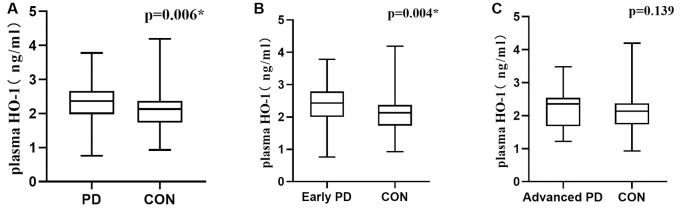
Plasma HO-1 levels in PD patients and control subjects. **(A)** Increased plasma HO-1 levels in the PD group compared with control subjects. **(B)** HO-1 levels in the PD group with early-stage were highly increased in the plasma compared with the controls. **(C)** Plasma HO-1 levels were elevated in the advanced PD group relative to the control group, but no statistically significant. PD, Parkinson’s disease; CON, normal controls; HO-1, Heme Oxygenase-1. The central line in each box indicates the median, box edges mark the first and third quartiles, and limits of the vertical lines show ranges. *p < 0.05.

Plasma HO-1 levels were significantly increased in PD patients compared with the control group [2.37 (1.98, 2.67) vs. 2.13 (1.73, 2.37) ng/ml; *p* = 0.006; Figure 1A]. Significantly higher HO-1 levels were observed in the participants with early PD than in the controls [2.43 (2.00, 2.79) vs. 2.13 (1.73, 2.37) ng/ml; *p* = 0.004; Figure 1B]. Although higher plasma HO-1 levels were detected in the subjects with advanced PD than in the healthy controls, this difference was not statistically significant [2.35 (1.68, 2.54) vs. 2.13 (1.73, 2.37) ng/ml; *p* = 0.139; Figure 1C]. In addition, differences in HO-1 concentrations based on H & Y stage were not significant [2.35 ± 0.89 vs. 2.37 ± 0.62 vs. 2.23 ± 0.58 vs. 2.15 ± 0.53 ng/ml; *p* = 0.787; Supplementary Figure 3].

There was no significant difference in LEDD levels among H & Y stages I-IV [400 (262.50, 631.25) vs. 400.00 (200.00, 600.00) vs. 450 (287.50, 600.00) vs. 400.00 (375.00, 737.50), *p* = 0.917; Supplementary Figure 2A]. The median LEDD in the 81 patients with PD was 400.00 mg. However, no significant correlation was observed between plasma HO-1 levels and LEDD (*r* = −0.015, *p* = 0.892; Supplementary Figure 2B).

**Figure 2:**
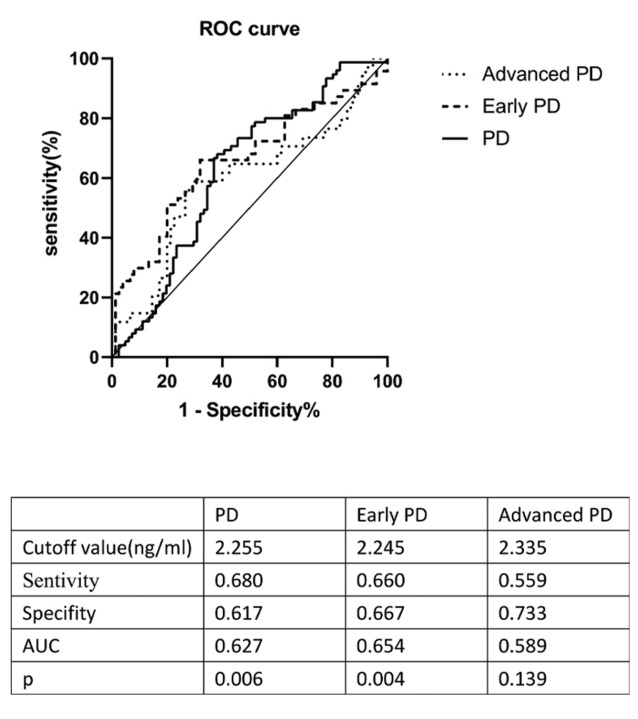
ROC curve for plasma HO-1 levels comparing PD group, early PD group, advanced PD group, and healthy control group. ROC curve, receiver operating characteristic curve; AUC, area under the curve; PD, Parkinson’s disease.

In order to evaluate the utility of HO-1 as a candidate biomarker of PD, an ROC curve was generated to distinguish PD patients in general from controls; the optimal cutoff value was found to be 2.255 ng/ml (sensitivity = 68%, specifity = 61.7%, AUC = 0.627, *p* = 0.006); the cutoff value for early-stage PD was 2.245 ng/ml (sensitivity = 66%, specifity = 66.7%, AUC = 0.654, *p* = 0.004). Plasma HO-1 levels did not differ between patients with advanced PD and the control group (sensitivity = 55.9%, specifity = 73.3%, AUC = 0.589, *p* = 0.139; Figure 2).

**Figure 3:**
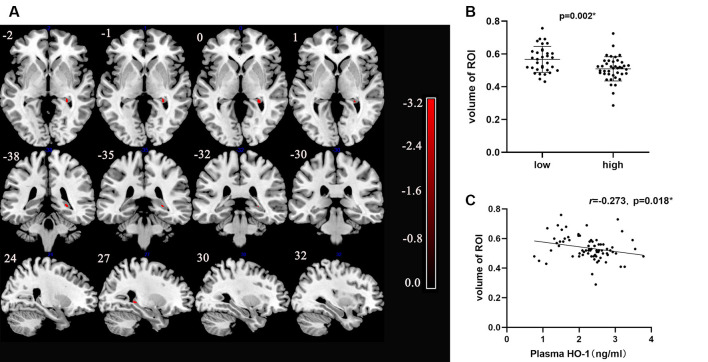
VBM analysis of differences in brain areas between low and high levels HO-1 groups. **(A)** The figure shows T1-weighted imaging-based VBM analysis of the whole-brain about series of sections images. Statistically significant reduction in clusters of right hippocampal concentration in high HO-1 PD group compared with that in low HO-1 PD subjects is shown. Only clusters comprising at least 10 suprathreshold voxels were included in the analysis and are shown in the figure. To help visualization, shaded red sections were added to highlight all clusters. Color-bar indicates the level of concentration by the depth of the color. **(B)** Comparison of the volume of two groups with different HO-1 levels. **(C)** Correlation between HO-1 level and right hippocampal volume. HO-1, Heme Oxygenase-1; VBM, Voxel-Based Morphometry. *p < 0.05.

Each volumetric image section in the high-HO-1 group showed a reduced area of the right hippocampus compared to the low-HO-1 group (Figure 3A). Brain regions with different volumes between the low- and high-HO-1 groups are summarized in Table 2. As shown in Figure 3B, the volume of the ROI was significantly higher in the low-HO-1 group than in the high-HO-1 group after the extraction of volumes in different brain areas (*p* = 0.002). Additionally, the correlation analysis indicated a weak but significant negative correlation between the ROI volumes and plasma HO-1 levels in the PD group (*r* = −0.273, *p* = 0.018; Figure 3C). There was no significant difference in MMSE scores between the low- and high-HO-1 groups [26.00 (23.00, 28.00) vs. 26.00 (24.75, 28.00), *p* = 0.533; Table 3]. However, the low-HO-1 group had lower language scores than the high-HO-1 group [3.00 (3.00, 4.00) vs. 4.00 (3.00, 4.00), *p* = 0.018; Table 3].

**Table 2:** Overview of VBM results (ROI analyses).

Region	Side	Cluster size	BA	MNI coordinates			*T*-value
				X	Y	Z	
Low > high							
Hippocampus	Right	20	37	27	−38	−18	−3.42
High > low*							

Differences of brain region comparing low and high HO-1 groups. *Means no areas were statistically significant different. BA, Brodmann’s Area; MNI, Montreal neurological institute; VBM, voxel-based morphometry; *T*-value is statistical *t* by two-sample *t*-test.

**Table 3:** MMSE and subscale score differences.

Items	Low	High	*p*-value
Orientation to time and place (10)	10.00 (9.00, 10.00)	10.00 (9.00, 10.00)	0.838
Immediate registration (3)	3.00 (3.00, 3.00)	3.00 (3.00, 3.00)	0.120
Divided attention (5)	4.00 (3.00, 5.00)	4.00 (2.75, 5.00)	0.803
Delayed recall (3)	3.00 (2.00, 3.00)	3.00 (1.75, 3.00)	0.331
Language (4)	3.00 (3.00, 4.00)	4.00 (3.00, 4.00)	0.018*
Following a three-step command (3)	3.00 (3.00, 3.00)	3.00 (3.00, 3.00)	0.118
Writing a sentence (1)	1.00 (0.00, 1.00)	1.00 (0.75, 1.00)	0.853
Copying a figure (1)	0.00 (0.00, 1.00)	0.00 (0.00, 1.00)	0.593
MMSE	26.00 (23.00, 28.00)	26.00 (24.75, 28.00)	0.533

Detailed cognitive function between low and high HO-1 levels groups. Data not conforming to a normal distribution were represented by median (interquartile range). HO-1, Heme Oxygenase-1; MMSE, Mini-mental State Examination. *p < 0.05.

## Discussion

Plasma is more accessible and frequently sampled than cerebrospinal fluid, and the assessment of plasma is inexpensive compared to PET-CT imaging techniques. In this study, we used plasma HO-1 levels to distinguish patients with PD from healthy controls in a large cohort. Concentrations of the HO-1 protein were significantly increased in patients with PD, particularly those in the early stage of the disease, compared to normal controls. VBM features are not suitable as markers, but they may improve our understanding of the potential pathophysiological mechanism underlying the differences in regional brain volume. Increased levels of the HO-1 protein were associated with a reduced right hippocampal volume.

HO-1 is the rate-limiting enzyme in the endoplasmic reticulum that converts the pro-oxidant heme into biliverdin, iron, and carbon monoxide in the presence of oxidative stress (Maines, 1988; Keyse and Tyrrell, 1989). HO-1 expression was observed in the neuromelanin-containing (dopaminergic) neurons of the substantia nigra compacta of postmortem brain tissues from patients with PD (Schipper et al., 1998). Considerable evidence implicates oxidative stress as a primary pathogenic mechanism underlying the death of most dopaminergic neurons in patients with PD (Blesa et al., 2015). The promoter of the HO-1 gene contains a heat shock element, and its expression is substantially upregulated in response to oxidative stress. The induction of HO-1 expression in redox tissues provides cytoprotection from oxidative damage through the production of bile pigments (bilirubin and biliverdin) with free radical-scavenging capabilities (Stocker, 1990). On the other hand, HO-1-mediated heme degradation releases free iron and CO, which exacerbates oxidative damage by producing free radicals within the mitochondrial compartment. However, little is known about the exact mechanism by which HO-1 participates in oxidative stress in patients with PD. Based on accumulating evidence, the HO-1 protein is present in peripheral body fluids, such as plasma (Bao et al., 2010; Signorelli et al., 2016; Kishimoto et al., 2018a,b; Kishimoto et al., 2020). Plasma HO-1 is leaked or secreted from epithelial and endothelial cells of different tissues that are subjected to oxidative stress. Additionally, the induction of HO-1 expression may be necessary and sufficient to provide protection after damage. Thus, plasma HO-1 levels are abnormal and elevated in patients with PD, a pathological condition, relative to the normal condition in healthy subjects.

As in previous studies (Mateo et al., 2010; Song et al., 2018), peripheral HO-1 levels were higher in patients with PD than in controls in the present study, and the difference was not affected by age, sex, or medication. Paradoxically, Schipper et al. (2000) did not observe a significant difference in plasma HO-1 levels between a small group of patients with PD and normal elderly subjects. A probable explanation for this discrepancy is the use of different methods to measure HO-1 levels in the two groups, along with the recruitment of subjects from different areas. A large cohort of patients with PD was used to investigate plasma HO-1 levels in the present study. Moreover, plasma HO-1 levels displayed moderate sensitivity and specificity in distinguishing PD patients from controls in the diagnosis of PD. In addition to the results discussed above, HO-1 concentrations were significantly increased in patients with early PD compared to controls without the disease, but there was no such difference between patients with advanced PD and healthy subjects. Our observations were consistent with those of a previous study (Song et al., 2018), where the level of HO-1 in patients with idiopathic PD peaked early and started decreasing as the disease progressed. As exemplified by a temporary elevation of particular chemicals in some patients with acute diseases, HO-1 protects against challenges or noxious stimuli resulting from oxidative damage in patients with early-stage PD. On the other hand, based on evidence from cell culture, animal model, and human neuropathology studies in the literature before this study, predominant HO-1 expression in astrocytes is considered a necessary and sufficient condition for excessive brain iron deposition, oxidative stress, mitochondrial dysfunction, and macroautophagy (Schipper et al., 2019). Hence, we hypothesized that the decreased level of HO-1 in patients with advanced-stage PD may be due to a mechanism by which the body defends against damage caused by HO-1 overexpression after the pathological features of PD have developed. Although HO-1 levels were markedly increased in the early stage of PD, plasma HO-1 levels must be measured in a larger number of patients with idiopathic PD.

The expression levels of HO-1 exhibited significant differences at various stages of PD. Therefore, we explored differences in the anatomical structures of brain regions in patients with different HO-1 levels. Interestingly, our VBM analysis showed a decrease in the volume of the right hippocampus in the group of PD patients presenting high HO-1 levels compared with the group presenting low HO-1 levels. According to Schipper et al. (1995) HO-1 was significantly overexpressed in hippocampal neurons and astrocytes in patients with AD relative to controls, as analyzed using immunoblotting, which confirmed an association between the hippocampus and HO-1 levels. Although this association was identified in patients with AD, both diseases are neurodegenerative diseases. GFAP.HMOX1 mice exhibit dysgenesis of the hippocampal dentate gyrus (Song et al., 2012). This model confirms the presence of hippocampal atrophy in mice overexpressing HO-1. Consistent with these results, we observed a reduction in hippocampal volume in patients with high peripheral plasma HO-1 concentrations. However, it is unclear why the volume reduction is limited to the right side of the hippocampus and whether the volumes of specific hippocampal subregions are decreased.

Also, we performed a simple cognitive function test (the MMSE) in PD patients with low and high HO-1 levels. The results showed that there was no significant intergroup difference in total MMSE scores. Several studies have reported that volumetric measurements of the hippocampus are the most significant predictor of AD among patients and that they may even precede the onset of clinical symptoms (Fox et al., 1996; Kaye et al., 1997; Jack et al., 1999). Additionally, hippocampal volume atrophy is a very sensitive indicator of cognitive impairment not only in people with AD but also in a broader population (Wolf et al., 2001). Taken together, the results imply that the reduction in hippocampal volume does not indicate impaired cognition. Perhaps this volume reduction occurs before the onset of cognitive impairment. The reason why the MMSE results were negative in this study may be that the clinical symptoms had not yet developed, although there were already structural changes. Regarding the specific sub-items of the MMSE, the reason for our results might be the limited specificity of the sub-items. More detailed data on cognitive function will be needed to compare the cognition of the two groups in future work. Our results also suggest that the correlation between hippocampal volume and HO-1 levels is complicated. Ultimately, plasma HO-1 is more promising than brain volumetric features as a marker of early PD, and increased plasma HO-1 is accompanied by a reduction in hippocampal volume, which can serve as a warning in the predementia stages.

The main strength of this study is that it is one of very few to measure peripheral plasma HO-1 levels in PD patients and control subjects in a sample size that allows statistical comparisons. Moreover, the anatomy of the brain was altered by HO-1 levels, advancing the current understanding of the underlying pathophysiological mechanism of HO-1. However, the study has some limitations. First, abnormal HO-1 levels have been observed in diseases of other organs, such as hypertension, diabetes, and cardiovascular disease (Bao et al., 2010; Signorelli et al., 2016; Kishimoto et al., 2018a,b, 2020). Although healthy controls with these complications were excluded from the study, these cofounders were still present in patients with PD. Second, plasma HO-1 concentrations in patients with atypical Parkinsonism, such as multiple system atrophy (MSA) and progressive supranuclear palsy (PSP), need to be measured in the future to determine the specificity of HO-1 as a marker of idiopathic PD. Third, larger sample size is warranted to analyze the role of HO-1 levels in predicting the progression of PD. In contrast to cross-sectional studies, a more accurate and appropriate approach would be to assess HO-1 levels and measure changes in the volumes of different brain structures in follow-up studies.

## Conclusion

Based on our findings, plasma HO-1 levels are a promising biomarker for diagnosing early PD and may assist clinicians in achieving unexpectedly good treatment outcomes by intervening in the early stage of the disease. Moreover, we observed a correlation between high HO-1 levels and GM volume in the hippocampus. Given the significance of reduced hippocampal volume, high HO-1 levels accompanied by a reduction in hippocampal volume in early PD may be a warning sign of cognitive impairment. Also, inhibiting HO-1 overexpression in response to nociceptive stimuli at a critical time in the development of PD might be an effective method for managing neurodegeneration.

## Data Availability Statement

The raw data supporting the conclusions of this article will be made available by the authors, without undue reservation.

## Ethics Statement

The studies involving human participants were reviewed and approved by Medical Ethics Committee of Henan Provincial People’s Hospital. The patients/participants provided their written informed consent to participate in this study.

## Author Contributions

WS, JM, and JZ completed the research design. WS performed the experiments. ZW, XS, and ML conducted the literature search and data collection. SH, SYH, ZZ, and DL performed the statistical analysis and interpretation of data. WS, JM, and JZ were responsible for writing the manuscript. All authors contributed to the article and approved the submitted version.

## Conflict of Interest

The authors declare that the research was conducted in the absence of any commercial or financial relationships that could be construed as a potential conflict of interest.

## Footnotes

^1^http://www.fil.ion.ucl.ac.uk/spm/software/spm8

^2^http://dbm.neuro.uni-jena.de

^3^http://www.alivelearn.net/xjview/ad.php

^4^http://restfmri.net/forum/rest_v12

^5^https://www.nitrc.org/projects/mricron

## Supplementary Material

Supplementary Figure S1 HO-1 levels were analyzed for all participants and stratified by age-group and sex-group. PD, Parkinson’s disease; HO-1, Heme Oxygenase-1. **p* < 0.05.

Supplementary Figure S2 The effects of medication. **(A)** The box plot between H and Y stages and LEDD. **(B)** Spearman correlation analysis for the relationship between LEDD and HO-1. LEDD, The equivalent daily dose of L-DOPA; H and Y stages, Hoehn and Yahr stages; HO-1, Heme Oxygenase-1. **p* < 0.05.

Supplementary Figure S3 Distribution of plasma HO-1 concentrations by H and Y stages. There were no significant differences in the distribution of plasma HO-1 concentrations by H and Y stages in PD patients (*p* > 0.05). H and Y stages, Hoehn and Yahr stages; HO-1, Heme Oxygenase-1. **p* < 0.05.
