# Supplementary figures and images for: Increased Plasma Heme Oxygenase-1 Levels in Patients With Early-Stage Parkinson’s Disease
Source: Front Aging Neurosci. 2021 Feb 12;13:621508. doi: 10.3389/fnagi.2021.621508 (PMC7906968; doi:10.3389/fnagi.2021.621508)

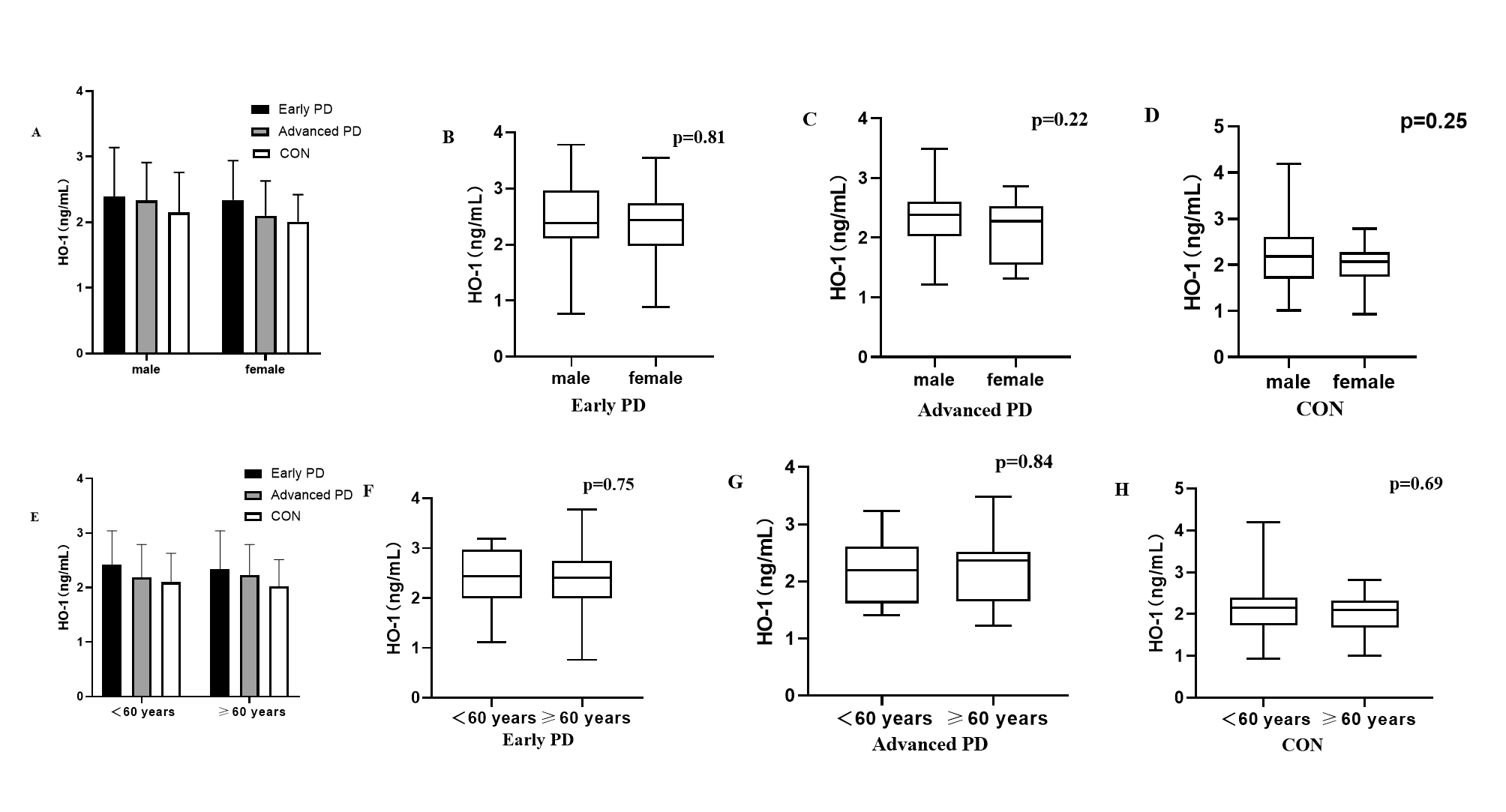

Supplement: Supplementary Figure S1 — HO-1 levels were analyzed for all participants and stratified by age-group and sex-group. PD, Parkinson’s disease; HO-1, Heme Oxygenase-1. *p < 0.05. [file Image_1.TIF]

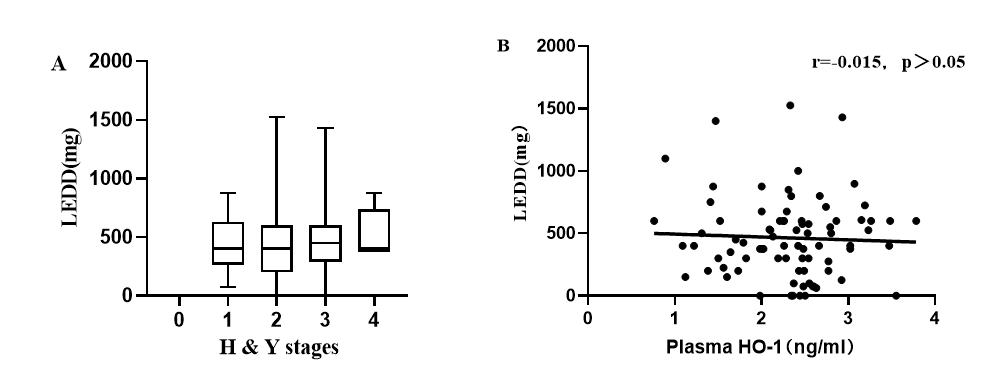

Supplement: Supplementary Figure S2 — The effects of medication. (A) The box plot between H and Y stages and LEDD. (B) Spearman correlation analysis for the relationship between LEDD and HO-1. LEDD, The equivalent daily dose of L-DOPA; H and Y stages, Hoehn and Yahr stages; HO-1, Heme Oxygenase-1. *p < 0.05. [file Image_2.TIF]

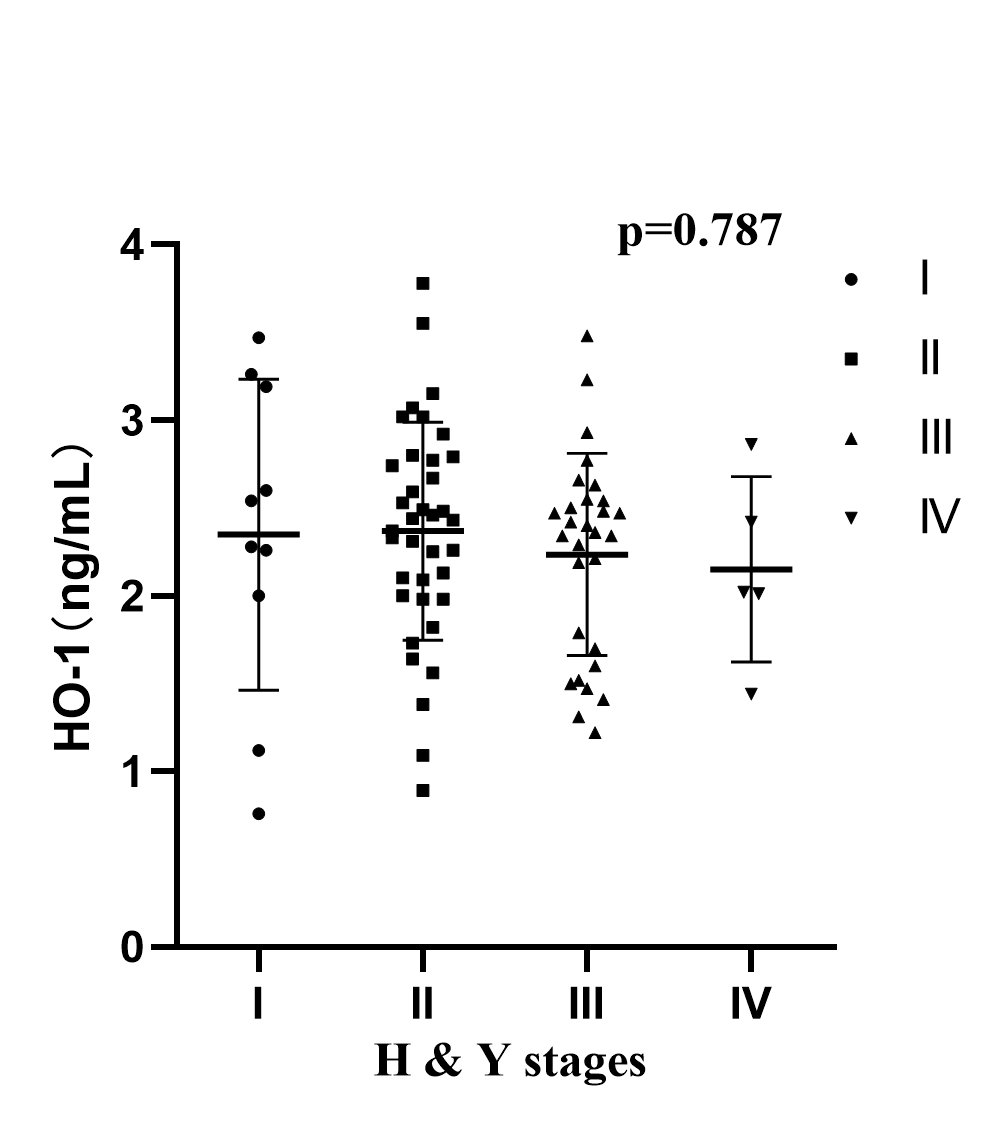

Supplement: Supplementary Figure S3 — Distribution of plasma HO-1 concentrations by H and Y stages. There were no significant differences in the distribution of plasma HO-1 concentrations by H and Y stages in PD patients (p > 0.05). H and Y stages, Hoehn and Yahr stages; HO-1, Heme Oxygenase-1. *p < 0.05. [file Image_3.TIF]
