# Supplementary material for: Increased Plasma Heme Oxygenase-1 Levels in Patients With Early-Stage Parkinson’s Disease
Source: Front Aging Neurosci. 2021 Feb 12;13:621508. doi: 10.3389/fnagi.2021.621508 (PMC7906968; doi:10.3389/fnagi.2021.621508)
Supplement: Supplementary file 4 [file Data_Sheet_1.docx]

**Supplementary Material**


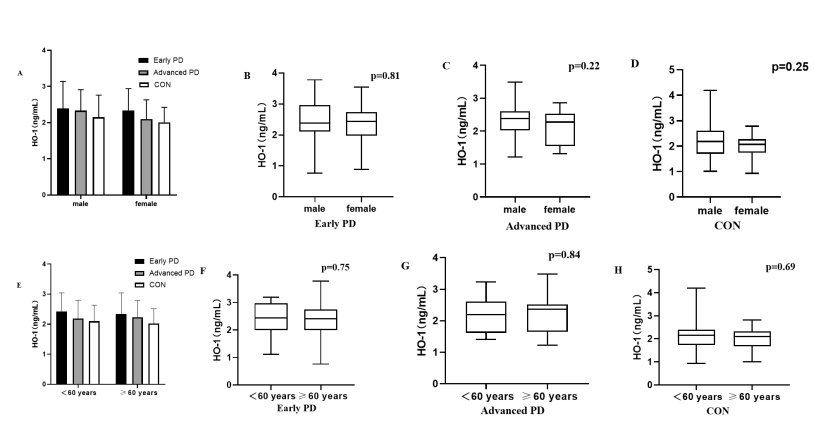


**FIGURE S1 |** HO-1 levels were analyzed for all participants and stratified by age-group and sex-group. PD, Parkinson’s disease; HO-1, Heme Oxygenase-1. **p*＜0.05.


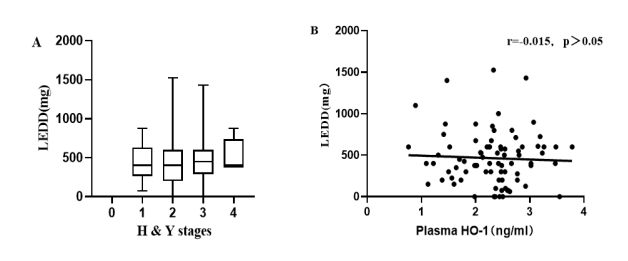


**FIGURE S2|** The effects of medication. **(A)** The box plot between H & Y stages and LEDD. **(B)** Spearman correlation analysis for the relationship between LEDD and HO-1. LEDD, The equivalent daily dose of L-DOPA; H & Y stages, Hoehn and Yahr stages; HO-1, Heme Oxygenase-1. **p*＜0.05.


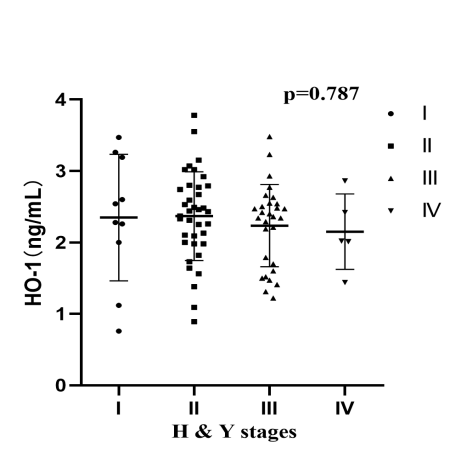


**FIGURE S3 |** Distribution of plasma HO-1 concentrations by H & Y stages. There were no significant differences in the distribution of plasma HO-1 concentrations by H & Y stages in PD patients (*p* > 0.05). H & Y stages, Hoehn and Yahr stages; HO-1, Heme Oxygenase-1. **p*＜0.05.
